# Nanomaterials as Inhibitors of Epithelial Mesenchymal Transition in Cancer Treatment

**DOI:** 10.3390/cancers12010025

**Published:** 2019-12-19

**Authors:** Marco Cordani, Raffaele Strippoli, Álvaro Somoza

**Affiliations:** 1 Instituto Madrileño de Estudios Avanzados en Nanociencia (IMDEA Nanociencia), 28049 Madrid, Spain; 2 Department of Molecular Medicine, Sapienza University of Rome, 00161 Rome, Italy; raffaele.strippoli@uniroma1.it; 3 National Institute for Infectious Diseases “Lazzaro Spallanzani” I.R.C.C.S., 00149 Rome, Italy; 4 CNB-CSIC-IMDEA Nanociencia Associated Unit “Unidad de Nanobiotecnología”, 28049 Madrid, Spain

**Keywords:** nanomaterials, nanomedicine, epithelial-mesenchymal transition, cancer metastasis, cancer therapy

## Abstract

Epithelial-mesenchymal transition (EMT) has emerged as a key regulator of cell invasion and metastasis in cancers. Besides the acquisition of migratory/invasive abilities, the EMT process is tightly connected with the generation of cancer stem cells (CSCs), thus contributing to chemoresistance. However, although EMT represents a relevant therapeutic target for cancer treatment, its application in the clinic is still limited due to various reasons, including tumor-stage heterogeneity, molecular-cellular target specificity, and appropriate drug delivery. Concerning this last point, different nanomaterials may be used to counteract EMT induction, providing novel therapeutic tools against many different cancers. In this review, (1) we discuss the application of various nanomaterials for EMT-based therapies in cancer, (2) we summarize the therapeutic relevance of some of the proposed EMT targets, and (3) we review the potential benefits and weaknesses of each approach.

## 1. Introduction

Epithelial-mesenchymal transition (EMT) is a highly dynamic multistep process implicated in a plethora of physio-pathological conditions, including cancer. In tumors, the acquisition of EMT has been demonstrated to provide the tumor cells with new migratory-invasive abilities, and, more recently, to mediate the acquisition of chemoresistance, which is linked to cancer stem cells (CSCs)-like features [1]. Importantly, the role of EMT in tumors may differ in different experimental systems and different tumors. EMT may be required for tumor initiation, for cell delamination from the primary tumor, for cell dissemination, and chemoresistance after therapy [2].

In this sense, there is growing evidence suggesting that targeting EMT can be used as a therapeutic approach itself, or to enhance the efficacy of other anticancer treatments [3,4,5].

Among the standard therapeutic strategies for cancer treatment (e.g., surgery, radiation, chemotherapy), the one that can be applied at all stages, including metastasis (stage 4), is chemotherapy since it is administered systemically [6]. However, it also presents a variety of drawbacks, such as low specificity, drug resistance, rapid drug clearance, and biodegradation, which can lead to treatment failures and related mortality [7]. In the last years, the use of drug delivery systems based on nanomaterials has improved the properties of a variety of bioactive therapies, including drugs, peptides, and antibodies [8,9,10]. Particularly, advances in material sciences have led to the development of different nanomaterials, which typically have sizes ranging from 5 nm to 200 nm and which properties can be exploited to treat cancer and/or be used as nanocarriers for drug delivery. These nanosystems are employed to improve the biodistribution of chemotherapeutic drugs and their accumulation at the target site, showing encouraging results in preclinical and clinical studies [11].

In this review, we summarized a plethora of studies describing the capability of nanostructures to inhibit EMT in cancer cells and in in vivo models, leading to cell death, chemosensitivity, and inhibition of cancer cell invasion and metastasis.

## 2. Epithelial to Mesenchymal Transition: From Physiology to Cancer

After being firstly described as a process allowing lens epithelium cells to invade a 3D collagen gel [12], eEMT has been increasingly characterized in many physio-pathological conditions, such as embryonic development, wound healing, organ fibrosis, and cancer progression [1]. EMT consists of a complex multistep reprogramming of the cell proteome, in which cells progressively lose their epithelial identity while gaining mesenchymal-like features.

EMT onset may be easily traced with conventional microscopy techniques: Under the appropriate stimulus, cells progressively lose their characteristic epithelial cell-to-cell junctions, such as adherent and tight junctions, and the apical-basal polarity. At the same time, front-rear polarity is acquired, as well as migratory and invasive abilities. Among epithelial markers, E-cadherin, a constituent of adherent junctions, is rapidly downregulated and is replaced by other pro-migratory plasma cadherins, such as N-cadherin, a phenomenon called ‘cadherin switch’. Similarly, intermediate epithelial filaments such as cytokeratin are replaced by mesenchymal counterparts, such as vimentin.

### 2.1. EMT-TFs

**Figure 1:**
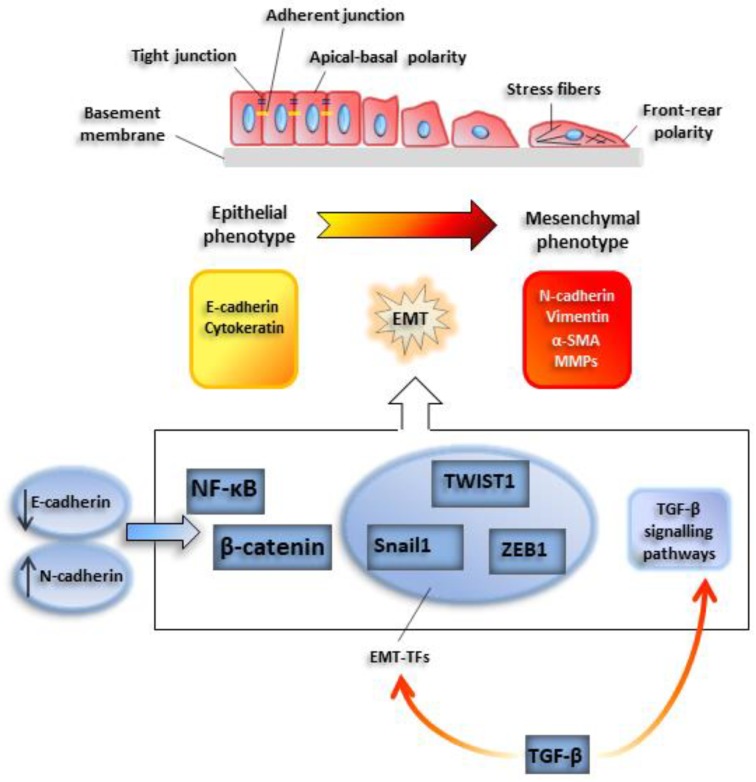
Outline of a typical epithelial-mesenchymal transition EMT program. Cells undergoing EMT progressively lose typical epithelial cell-to-cell junctions, such as adherent and tight junctions, and the apical-basal polarity. At the same time, cells acquire a front-rear polarity, while gaining migratory and invasive abilities. Among epithelial markers, E-cadherin, a constituent of adherent junctions, is rapidly downregulated and is replaced by N-cadherin. This phenomenon is called ‘cadherin switch’. Similarly, epithelial intermediate filaments such as cytokeratins are replaced by mesenchymal counterparts, such as vimentin. Also, smooth muscle actin (α-SMA) is de novo induced. E-cadherin downregulation facilitates the activity of transcription factors relevant for the induction of the EMT program, such as nuclear factor kappa B subunit 1 (NF-κB) and β-catenin. Snail1, TWIST1, and ZEB1 are EMT-activating transcription factors which lead activation of EMT program.

The loss of E-cadherin is mediated by basic helix loop helix (bHLH) or zinc-finger transcription factors, such as Snail-1, Twist-related protein 1 (TWIST1), and zinc finger E-box binding homeobox 1 (ZEB1) [1]. These proteins are considered master genes of EMT or EMT-activating transcription factors (EMT-TFs), since their expression is sufficient to promote EMT induction [13]. The activity of EMT-TFs often implicates the involvement of epigenetic factors, such as histone deacetylases (HDACs), methyl-transferases, and demethylases, such as lysine demethylase 1 (LSD1) [14] (Figure 1).

Besides the EMT-TFs, different TFs involved in other cellular processes play a critical role in EMT induction. For instance, NF-ĸB, a TF activated during inflammation, has been demonstrated to control also SNAIL-1-E-cadherin expression [15,16]; the Wingless-type MMTV integration site WNT1-β catenin pathway links the stability of cellular junctions to the repression of EMT program in tumors [17].

### 2.2. Extracellular Inducers of EMT: Role of TGFβ

**Figure 2:**
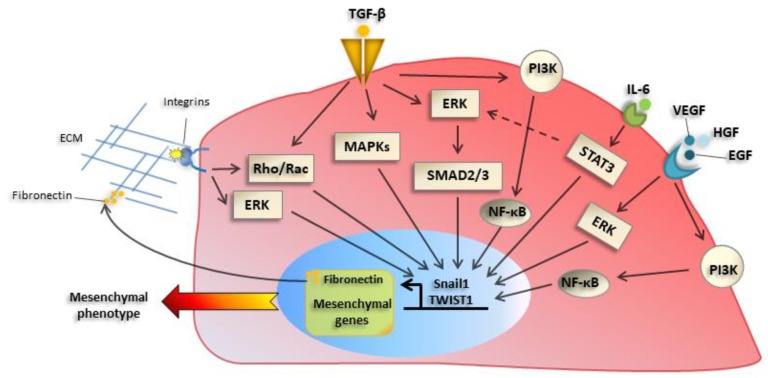
Main molecular mechanisms underlying EMT activation. Transforming growth factor-β (TGF-β) receptor activation induces a classical mother against decapentaplegic (SMAD) pathway, as well as ‘nonclassical’ pathway, such as mitogen-activated protein kinase (MAPKs), Phosphoinositide 3-kinase (PI3K)- mammalian target of rapamycin (mTOR), or Rho guanosine triphosphatase (GTPases), which induce EMT-TFs such as Snail1 and TWIST1. Besides their activity as transcriptional regulators, they control the expression of mesenchymal genes and extracellular matrix (ECM) proteins, such as fibronectin and collagens, whose production is increased during EMT. The alteration of ECM composition causes changes in expression and activation of integrins, which promote migratory proprieties of cancer cells. Integrin signaling in turn sustains EMT induction through extracellular signal–regulated kinase (ERK) and Rho GTPases signaling pathways. Tyrosine receptor ligands (EGF, HGF) and cytokines, such as IL-6 and VEGF, are other factors involved in EMT activation through ERK, PI3K, and NF-κB pathways.

Among the plethora of extracellular EMT inducers, proteins of the transforming growth factor β (TGFβ) family play a central role, inducing EMT-TFs expression. Besides their activity as transcriptional repressors, EMT-TFs such as Snail1, are direct inducers of fibronectin (FN), which is part of the extracellular matrix (ECM) proteins whose production is increased during EMT [16]. The changes in ECM composition, as well as soluble factors, cause increased expression and activation of integrins, especially of β1 and β3 families. This, in turn, has a role in promoting an adhesive/migratory phenotype, as well as changes in proliferation/survival [18]. Integrin signaling cooperates with signals elicited by tyrosine kinase or TGFβ induced pathways, leading to increased adhesion/motility. Besides TGFβ, other extracellular factors such as tyrosine receptors ligands including Epidermal growth factor, (EGF) and Hepatocyte growth factor (HGF) and cytokines such as Interleukin6 (IL-6) [19] and Vascular endothelial growth factor (VEGF) promote EMT in different experimental systems (Figure 2).

### 2.3. Noncoding RNAs

Besides the processes mentioned above, the discovery and characterization of small noncoding RNAs, such as microRNAs (miRNAs), or more recently of long noncoding RNAs added a new layer in the study of molecular mechanisms undergoing EMT induction. In this sense, molecular circuits between miRNAs and EMT-TFs have been elucidated, such as that one involving miR-200 and ZEB1 [20]. Other examples involve miRNA-29, miRNA-378b, and miRNA-26a-5p, which are direct regulators of EMT and fibrosis through collagen expression by several mechanisms, such as by inhibiting sirtuin-6 and the autophagic protein ULK-1 [21,22,23].

Interestingly, EMT induction is a dynamic and multistep process, and in the first phases it may be reversed. In this sense, under the appropriate stimulus, cells in a mesenchymal state may progressively lose their mesenchymal features, while recovering an epithelial identity. This process is known as mesenchymal to epithelial transition (MET). This process is characterized by the re-expression of epithelial proteins, such as E-cadherin and cytokeratins, and the downregulation of protein characteristic of the mesenchymal phenotype, such as EMT-TFs, N-cadherin, and vimentin [24,25].

### 2.4. Specificities of Tumor EMT

Although EMT is involved in a myriad of physio-pathological cellular processes, three main different types of EMT have been described, particularly those: (1) Occurring during embryogenesis, (2) occurring during chronic fibrotic pathologies, and (3) taking place during tumor progression [26]. The first type is essential for physiologic implantation, development, and embryogenesis. The second type occurs during chronic inflammatory-fibrotic pathologies and is associated with fibrosis and organ dysfunction. The third type is characteristic of carcinomas, i.e., cells that have already undergone rounds of genetic-epigenetic changes and show substantial alterations in proliferation/cell death mechanisms.

Thus, tumor-related EMT is now considered one of the primary mechanisms of tumor progression [13]. This process has some specificities concerning other forms of EMT: For instance, cancer cells most often undergo an incomplete or partial EMT [26]. Also, EMT features may be acquired by non-epithelial tumors, such as sarcomas. Similarly to nontumor cells, the induction of the EMT program is promoted by the expression of EMT-TFs [27,28]. Interestingly, although genetic alterations are always present in tumors, EMT-TFs are rarely mutated in cancers.

Cells under EMT have enhanced motility and invasiveness, allowing them to break the basal lamina and reach the stromal areas, which are rich in hematic and lymphatic vessels. The increased migration-invasion is mediated by the formation of invadopodia, which are actin-rich protrusions expressing at the cell edges gelatinases or matrix metalloproteases (MMPs), such as Membrane type 1 (MT1)-MMP or MMP2/MMP9. Epithelial cells generally do not express these enzymes, which can degrade the ECM, and their acquisition is a critical step towards a malignant phenotype.

Tumor cells undergoing EMT generally produce an increased amount of ECM proteins, such as collagens, Fibronectin (FN), and vitronectin, and often cooperate with the cells of the local niche (such as cancer-associated fibroblasts, (CAFs), and tumor-associated macrophages, (TAM)) to remodel surrounding tissues to facilitate angiogenesis and cellular invasion, and to modulate local immunity. Besides facilitating invasion and metastasis, EMT in tumors increases survival and chemoresistance. EMT induction allows tumor cells to escape anoikis (death upon cell detachment) and to survive while flowing through hematic and lymphatic vessels, promoting metastasis. This process is generally related to the repositioning of an invading tumor cell to a new proper niche and is often associated with the rescue of an epithelial-like phenotype, a process called mesenchymal to epithelial transition (MET). MET induction is mediated by the different ECM composition of the new niche but also by local soluble factors. Chemoresistance is related to the expression of EMT-TFs involved in the survival of the cells, such as Snail-1, -TWIST1, and ZEB1, even in the absence of increased invasive/metastatic abilities [29]. To this respect, tumor cells undergoing EMT may acquire a profile similar to that of cancer stem cells (CSC). However, the relationship between EMT and stemness is still unclear [13].

Thus, since the EMT process is crucial for cancer-enhanced invasion, metastasis, chemoresistance, and survival, it is now the target of different experimental therapeutic approaches [3,4,5]. However, even though the role of EMT in the pathogenesis of cancer transformation has been proven in vitro and in vivo, its translation to the clinic has failed in most cases. One possible explanation may reside in the lack the optimal systems for delivery of therapies targeting EMT. In this review, the recent applications of nanomaterials to target EMT in cancer will be discussed.

## 3. Nanomedicine in Cancer Therapy

**Figure 3:**
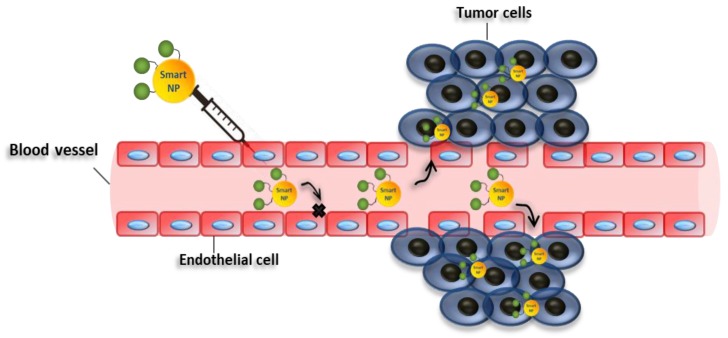
Enhanced permeability and retention effect. Schematic representation showing enhanced permeability and retention of nanoparticles in tumor vessels. Smart nanoparticles can passively accumulate in weak blood vessels, typical of tumor tissues. In this case, drug may be released in the extracellular matrix and then diffuse through the tissue.

The recent advances in nanotechnology offer many tools to fight cancer, which can be exploited to bypass the difficulties usually encountered with traditional drugs [30]. For instance, nanostructures can be used as nanocarriers, which may passively accumulate in weak blood vessels, typical of tumor tissues, due to the well-known enhanced permeability and retention effect (EPR) [31] (Figure 3). Nanocarriers can also be conjugated with molecules with selective binding capabilities, exploiting active targeting mechanisms [32] to improve further the selectivity of the therapy. Besides, nanocarriers can be employed to improve the pharmacokinetic and pharmacodynamic properties of drugs, thus, improving their therapeutic efficacy [31,33]. Moreover, the release of the therapeutic molecules may be regulated by internal stimuli at the target sites, such as pH, redox potential, ionic strength, enzymes, and other stresses [34], or external stimuli, such as temperature, light, ultrasound, magnetic force, and electric fields [34]. These strategies can be exploited to increase the selectivity of the system, decreasing the undesirable effects of the drugs [35]. Also, nanomaterials can have a cytotoxic activity by themselves, and/or increase the efficacy of standard chemotherapies.

Depending on their structural and chemical-physical properties, nanomaterials are divided into several classes, such as liposomes, carbon-based, polymers, metals, metals oxide, and ceramics. In most cases, these nanomaterials are employed as nanocarriers to release therapeutic molecules, such as drugs, proteins, or nucleic acids [36,37,38,39,40,41,42,43,44,45,46,47].

## 4. Targeting EMT with Nanoparticles for Cancer Therapy

In this section, we comment on the recent studies describing the ability of nanomaterials to inhibit the EMT process in cancer cells, which can be employed to modulate several key features such as migratory/invasive capabilities, stem cell phenotype, and tumor progression (Table 1). 

### 4.1. Modulating EMT with Unmodified Nanomaterials

In addition to the advantages of using nanomaterials as nanocarriers [98,99], their inherent properties can also be exploited for therapeutic purposes. In this sense, gold and metal oxide nanoparticles have shown remarkable capabilities in the inhibition of the EMT process.

#### 4.1.1. Gold Nanoparticles

Gold nanoparticles (AuNPs) have been largely exploited in biomedicine as drug delivery systems, because of their low toxicity, biocompatibility, and stability [100]. In this regard, the surface of AuNPs can be modified with a variety of bioactive molecules, such as chemotherapy drugs, nucleic acids, and proteins.

It has been reported that unmodified AuNPs inhibit cell proliferation and metastasis and enhance the sensitivity to cisplatin and gemcitabine in in vitro and in vivo models of pancreatic and ovarian cancer by reverting the mesenchymal phenotype of cancer cells to the epithelial one [48,49,50]. This occurs by reducing the expression of key stem cell markers as well as EMT-related markers and by preventing the activation of Akt, NF-ĸB, and MAPK signaling axis, which are crucial for EMT, stemness, and drug resistance [48,49,50].

Another study revealed that AuNPs treatment was able to downregulate EMT in melanoma tumors by reducing the expression of some EMT proteins such as vimentin, E-cadherin, and MMP2 [51]. Consequently to EMT modulation, these nanomaterials lead the normalization of the tumor vasculature, mitigate the hypoxia in melanoma tumor, suppress the migration of Human umbilical vein endothelial cells (HUVECs) and B16F10 cells, and decrease melanoma tumor metastasis in vivo [51]. Besides, the inherent properties of metallic nanoparticles can be exploited for hyperthermia-based approaches [101], where body tissues undergo high temperatures to injure cancer cells or to sensitize them to chemo/radiotherapy. In this regard, hyperthermia has been shown to inhibit EMT in tongue squamous cell carcinoma [102], pancreatic adenocarcinoma cells [103,104], and hepatocellular carcinoma [105,106], resulting in inhibition of cancer cell invasion and sensitizing gemcitabine-resistant pancreatic adenocarcinoma cells to gemcitabine.

In the case of metallic nanoparticles, it is possible to produce local heating at the nanoparticle-containing cells without hurting the neighboring healthy tissues, when irradiated by a laser at a wavelength near their plasmon-resonant absorption band [107]. In this regard, gold nanorods (AuNRs) have been used in plasmonic photothermal therapy (PPTT) to block EMT, leading to a significant reduction in cancer cell collective migration [52].

Another study reported that the combined treatment of Polyethylene glycol (PEG)-coated AuNPs and PPTT was able to inhibit EMT by preventing the activation of the PI3K/Akt pathway in glioblastoma and lung cancer cells [53]. This co-treatment also suppressed tumor growth in xenograft mice models, indicating a direct link between EMT and cell death in solid tumors [53].

#### 4.1.2. Metal Oxide Nanoparticles

Titanium dioxide nanoparticles (TiO_2_NPs) have been exploited to deliver a variety of therapeutic molecules, including chemicals, antibodies, or modified nucleic acids for cancer therapy [108]. A recent study revealed that TiO_2_NPs suppress the EMT process by blocking TGFβ signaling in cancer epithelial cells, leading to inhibition of cell migration without exhibiting cytotoxicity [54].

In particular, TiO_2_NPs induce lysosomal degradation of the TGFβ receptor complex (TβRI/II), and thereby downregulate the expression of TGFβ-target genes. TiO_2_NPs and silicon dioxide (SiO_2_NPs) were able to suppress ECM production, blocking the effect of TGFβ in cells implicated in liver fibrosis [55].

Zinc oxide nanostructures (ZnO-NSts), such as nanoplates (NPls), nanorods (NRs), nanosheets (NSs), and nanoflowers (NFs) have shown to play a role in the control of cancer growth and apoptosis, but the mechanisms implicated remain unclear [109,110].

In this regard, a novel study reports that these ZnO-NSts nanostructures downregulate EMT markers exerting cytotoxic effect against glioblastoma, lung, and thyroid cancer cells [56]. The inhibition of the mesenchymal phenotype by these drugs led to the suppression of cancer invasiveness and the induction of cell death due to oxidative stress and caspase-dependent pathways [56].

Hence, unmodified metal oxide nanostructures could help the control of cancer growth and metastasis by regulating EMT and represent a new promising therapeutic tool.

### 4.2. Delivery of Small Molecules to Inhibit EMT

A variety of nanoparticles have been successfully employed to deliver small molecules, such as natural products and/or synthetic drugs, to suppress or reverse EMT via the inhibition of associated signaling pathways in various cancer cells and in in vivo models (Table 2).

#### 4.2.1. Natural Products

The development of drugs and therapeutic molecules derived by natural products represents an exciting opportunity for the treatment and prevention of cancer. Interestingly, in recent years, several studies reported the use of nanomaterials as carriers of natural substances, alone or in combination with chemotherapy, to treat cancer by suppressing EMT and the associated TFs and signaling pathways [57,58,59,60,61,62,63,64,65,66,67]. The main natural products employed are commented on below.

#### 4.2.2. α-Mangostin and Anthothecol

Among the plethora of plant-derived compounds having therapeutic proprieties, α-mangostin and anthothecol have elicited particular interest for antioxidant, antimicrobial, anticancer, and anti-inflammatory properties [113,114,115,116,117]. The α-mangostin was found to suppress EMT by inhibiting MMP2 and MMP9 and increasing E-cadherin expression by downregulating the PI3K/Akt pathway [118].

However, the in vivo application of most polyphenols was limited due to poor bioavailability, poor solubility, and low pharmacokinetic profiles [119].

To overcome these obstacles and increase the efficacy, α-mangostin and anthothecol have been encapsulated into the core of poly (D, L-lactic-co-glycolic acid) (PLGA) nanoparticles (Mang-NPs and Antho-NPs) and evaluated as a therapeutic approach in pancreatic cancer stem cells [57,58].

PLGA is largely used for drug delivery because it presents excellent biodegradable and biocompatible properties [120]. Mang-NPs and Antho-NPs were found to inhibit EMT by upregulating E-cadherin and inhibiting N-cadherin and transcription factors Slug, Snail, and Zeb1, as well as by downregulating pluripotency maintaining factors Nanog, c-Myc, and Oct4 and components of Shh pathway [57,58]. EMT suppression by these nanomaterials resulted in the inhibition of proliferation, migration, and development of pancreatic cancer cells as well as the reduction of malignant transformation and metastasis in in vivo models [57,58].

#### 4.2.3. Wedelolactone

Wedelolactone (Wdl) is another plant-derived polyphenolic compound that has been found to inhibit androgen-independent prostate cancer [121], endometrial and ovarian cancer cell growth, [122] and suppress the EMT of alveolar epithelial cells [123]. In order to increase its effectiveness and diminish toxic side effects, Wdl has been encapsulated into PLGA nanoparticles (nWdl) and used as anticancer therapy in breast cancer [59]. These polymeric nanocomplexes prevented EMT, reduced cell migration and invasion, and diminished the percentage of breast cancer stem cells (brCSCs) in MDA-MB-231 cells [59]. Intriguingly, nWdl also sensitized brCSCs to paclitaxel, reducing the percentage of ALDH+ brCSCs, and suppressed tumor growth in mice [59].

#### 4.2.4. Salinomycin

Salinomycin (SAL) is a monocarboxylic polyether antibiotic isolated from *Streptomyces albus* strain with activity against pancreatic cancer stem cells [124,125].

Interestingly, SAL also was found to reduce EMT-mediated multidrug resistance in gastric cancer [126]. However, the use of NPs offer several advantages for SAL delivery, including improved solubilization, increased intratumor accumulation through EPR effect, high stability, and low side effects [127].

In a recent study, SAL was delivered in an orthotopic model of pancreatic cancer using PLGA nanoparticles [60]. SAL-loaded PLGA NPs led to EMT inhibition and apoptosis induction in AsPC-1 pancreatic cancer cells as well as reduction of tumor growth in AsPC-1-luc orthotopic pancreatic model [60].

In another study, SAL was delivered using polymeric micelles and was shown to repress EMT in lung cancer, leading a reduction of migration capability of A459 lung cancer cells without interfering with cell proliferation [61].

#### 4.2.5. Gallic Acid

Gallic acid (GA) is a potent antioxidant discovered in plants and many fruits and is beneficial in inhibiting metastasis in glioma [128], gastric [129], and prostate cancer cells [130]. Some reports showed that GA shows efficacy against EMT-related markers, and it is a potential therapeutic agent in pulmonary fibrosis in mice [131].

Different studies highlighted that silver nanoparticles (Ag-NPs) possess a high therapeutic potential against a plethora of cancer cells by modulating autophagy and acting as cytotoxic agents by itself [132]. In addition, it has been reported that these nanomaterials can act as nanocarriers to deliver therapeutic molecules, such as GA, hampering EMT, and thus reducing the metastatic potential of A459 lung cancer cells [62].

#### 4.2.6. Curcumin

Curcumin, a phytochemical derived from *Curcuma longa*, has been largely exploited in combination with traditional chemotherapeutics to increase their anticancer efficacy [133]. Besides, recent reports showed that curcumin suppresses EMT and metastasis in various tumor cells [134,135,136].

However, the therapeutic efficacy of curcumin is limited by its poor bioavailability and absorption, rapid metabolism, and rapid systemic elimination [137]. Therefore, the use of certain nanoparticles represents an effective way to improve the bioavailability and pharmacokinetics, thus enhancing its therapeutic effect, internalization, and tumor targeting [137].

Several studies reported the potential anticancer efficacy of selenium nanoparticles (Se-NPs) on prostate, liver, cervical, and breast cancer cells [138,139,140].

In this regard, curcumin-loaded selenium nanoparticles (Se-Cu-NPs), alone and/or in combination with doxorubicin (DOX), were found to inhibit EMT and reduce inflammation, metastasis, and chemoresistance in colon cancer. This occurred consequently to the alterations of crucial EMT-TFs, such as Snail and NF-ĸB [63,64]. Notably, Se-Cu-NPs reduced the tumor mass, enhancing the mean survival time of tumor-bearing Ehrlich’s ascites carcinoma (EAC) mice [63,64].

#### 4.2.7. Quercetin

Quercetin (Qu) is a bioflavonoid that possesses anticancer properties since it was found to modulate EMT, proliferation, survival, and differentiation of tumor cells [141,142,143]. However, the therapeutic efficacy of Qu is limited by its poor solubility in water. In order to enhance Qu effectivity, a gold nanoparticle-based drug delivery system for Qu (AuNPs-Qu-5) has been formulated and employed against breast cancer cells. These nano-conjugates were found to suppress EMT, leading to the reduction in angiogenesis, tumor growth, and metastasis in MCF-7 and MDA-MB-231 breast cancer cell lines. Notably, treatment with these nanoparticles led to tumor regression in 7,12-dimethylbenz(a)anthracene (DMBA)-induced mammary carcinoma in Sprague–Dawley rats [65].

Other approaches have exploited the use of liposomes, which present many advantages as nanocarriers, presenting excellent biocompatibility and improving the solubility, stability, and pharmacokinetic properties of the drugs [144]. In this regard, it has been reported that esophageal cancer stem cells exposed to nanoliposomal quercetin (nLQ) showed inhibited EMT and the modulation of a variety of EMT-related proteins, such as HDAC1 and E-cadherin [66]. The reversion of the mesenchymal towards the epithelial phenotype reflects the capacity of these nanoparticles to act as a cancer therapeutic agent. 

#### 4.2.8. Epigallocatechin Gallate

Epigallocatechin gallate (EGCG), a compound extracted from green tea, has been proven to have multiple anticancer, anti-inflammatory, anti-EMT, and anti-oxidant proprieties [145,146,147]. Despite its ability to act as an excellent anticancer drug, EGCG displays some limitations related to stability, bioavailability, and metabolic conversion under physiological conditions [148,149].

In order to address these issues, an innovative coating membrane where EGCG is coordinated with ferric ions to generate epigallocatechin gallate/iron nanocomplexes (EIN) [67] has been reported. EIN were used to coat mesoporous silica nanoparticles (MSN) and PEG-PLA micelles (Mic) to form MSN@EIN and Mic@EIN, respectively. MSN@EIN and Mic@EIN displayed ideal biocompatibility, low cytotoxicity, and increased intracellular drug concentration leading to EMT inhibition when delivered in breast cancer cells. These nanostructures were able to eliminate EMT-type cancer cells in in vivo models, to decrease cancer cell migration, and to prevent drug resistance [67]. Moreover, it has also been described that MSNs coated with epigallocatechin gallate reduced EMT and tumor metastasis when delivered in in vivo models of breast cancer [67].

### 4.3. Synthetic Drugs

Different nanomaterials have been exploited to deliver synthetic chemotherapeutics and chemicals inside cancer cells reverting EMT and chemoresistance, and prevent metastasis in a plethora of tumor types [68,69,70,71,72,73,74,75,76,77,78].

#### 4.3.1. Etoposide

Etoposide (VP16) is a semisynthetic phytophthora toxin widely used as a chemotherapeutic agent for the treatment of several cancers [150,151]. However, many studies showed that VP16 fails against glioma stem cells (GSCs), resulting in enhanced tumor metastasis and recurrence [152,153].

Many factors were correlated with VP16 resistance to CSCs, including poor water solubility, metabolic inactivation, low bioavailability, enhanced drug extrusion by ATP-binding cassette multidrug transporters, and increased rates of DNA repair [154,155,156].

In order to bypass these obstacles and improve the clinical applications, layered double hydroxide (LDH) nanocomposites (L-V) have been complexed with Etoposide (VP16). Wang and colleagues showed that these nanocomplexes were able to inhibit EMT modulating EMT-related signaling pathways, leading to the elimination of glioma stem cells and reduction of tumor growth [68]. In in vivo GSCs, xenograft mice model L-V could overcome drug resistance, leading to a reduction in tumor growth [68].

#### 4.3.2. ADH-1

ADH-1 (N-AC-*CHAVC*-NH_2_), a cyclic pentapeptide obtained from the His-Ala-Val (HAV) site of N-cadherin, is a potent antagonist of N-cadherin-mediated adhesion and invasion. In this regard, it was reported that ADH-1 was able to inhibit N-cadherin dependent tumor progression in vitro and in vivo [157,158,159]. As mentioned above, liposomes are excellent, easily modifiable nanocarriers for anticancer drugs, which enhance their therapeutic efficiency and represent promising cancer treatments in the future.

In a recent study, ADH-1 peptide-modified liposomes (A-LP) were prepared to facilitate the delivery of the chemotherapeutic drug paclitaxel (PTX) to tumor cells undergoing EMT [69].

Intriguingly, these liposomal nanocomplexes were able to inhibit the EMT process and breast cancer cell migration and restore chemosensitivity, exhibiting strong antitumor efficacy in vivo [69]. 

For the treatment of cancer through EMT suppression, the use of several systems based on mesoporous silica (MSNs) and mesoporous titanium (NTNs) nanoparticles functionalized with hyaluronic acid (HA), DOX, and ADH-1 [70,71] has been reported. These multifunctional nanocarriers, alone and/or in combination with photodynamic therapy (PDT), led to EMT suppression in A549 lung carcinoma cells, thus promoting drug sensitivity and inhibition of cell migration, and reduced the metastatic potential [70,71].

#### 4.3.3. Dexamethasone

Glucocorticoids (GCs) are a family of steroid hormones acting through the glucocorticoid receptor (GR), a member of the nuclear receptor superfamily [160]. Recent studies report that GCs can block EMT of mink lung epithelial cells and estrogen receptor (ER)-negative breast cancer cells [161,162]. Synthetic GCs are largely used as anti-inflammatory drugs [163] and dexamethasone (Dex) was found to reduce EMT in colon cancer cells as well as in human peritoneal mesothelial cells [164,165]. However, long-term systemic exposure to Dex causes a variety of adverse effects [166], and novel strategies are needed to deliver Dex in cancer cells.

In this regard, a study by Agarwalla et al. reports that AuNPs modified with dexamethasone (DEXA) and withaferin-A were able to inhibit EMT in tumor cells preventing metastasis [72]. These nanostructures led to glucocorticoid receptor-dependent selective cytotoxicity, inhibited melanoma growth in mice, and suppressed mice mortality [72].

#### 4.3.4. Arsenic Trioxide

Arsenic trioxide (ATO) exhibits anticancer effects by degrading the oncogenic EVI1 protein, an interactor of Snail and HDAC1 active in E-cadherin repression and EMT induction [167]. 

Additionally, several studies showed that ATO could suppress EMT in gastric and liver cancer by different mechanisms [168,169]. Despite some successes as anti-EMT agent, ATO has poor bioavailability and causes adverse reactions that limit its clinical application in treating human cancers [170]. Hence, novel approaches to reduce off-target effects and improve bioavailability are required.

In this sense, zinc arsenite (ZnAs) NPs have been exploited as a molecular scaffold to deliver ATO in nasopharyngeal carcinoma (NCO) cells and hepatocarcinoma (HCC) tumor models [73,74]. ATO-loaded zinc arsenite (ZnAs) nanocomplexes inhibited EMT process via SHP-1/JAK2/STAT3 pathway and by reducing the expression of EMT-related proteins, leading to reduced tumor growth in vivo [73,74].

#### 4.3.5. Rhenium-188

Rhenium-188 (^188^Re) is a high-energy β-particle radionuclide obtained from an alumina-based tungsten-188 (^188^W)/^188^Re generator [171]. The short average penetration distance of β-particles in soft tissues makes ^188^Re an ideal radionuclide for tumor ablation with reduced activity on the surrounding normal tissues [172].

Interestingly, the radiopharmaceuticals generated through the conjugation of liposomes with radionuclide ^188^Re are considered useful for theranostics purposes in cancer diseases by enhancing internal circulation without acute toxicity through the EPR effect [173].

Polyethylene glycol (PEG)-coated ^188^Rhenium-liposome has been shown to inhibit EMT in murine models of ovarian tumors [75] and human head and neck squamous cell carcinoma (HNSCC) [76,77], exhibiting killing efficacy and tumor growth delay through induction of *let7* miRNA and glycolysis inhibition.

**Figure 4:**
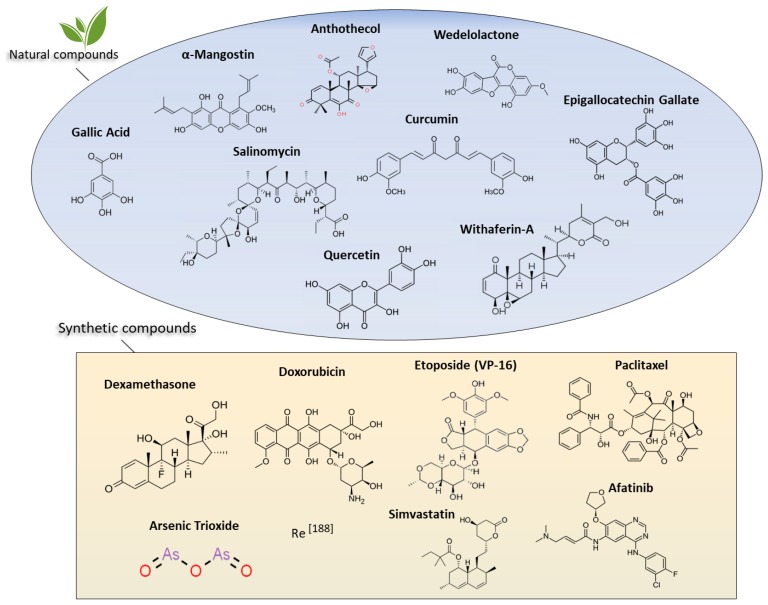
Chemical structures of natural substances and synthetic therapeutics delivered by nanomaterials active in tumor EMT inhibition discussed along this review.

The therapeutics delivered by nanostructures commented on here are represented in Figure 4.

### 4.4. Nanoparticles for Nucleic Acids Delivery to Revert EMT Phenotype

Therapeutic nucleic acids have been largely explored as anticancer tools [174], particularly RNA-based derivatives, such as small interfering RNAs (siRNAs), and micro RNA (miRNA) mimics and antagomirs (see below). Although their use in targeting EMT is promising, nucleic acids have to face important obstacles limiting their therapeutic effect, including nucleic acid stability and successful delivery in vivo. In this regard, a variety of nanocarriers have been developed to address these issues, opening novel therapeutic opportunities.

#### 4.4.1. Delivery of SiRNAs Against EMT TFs and Other EMT-Related Genes

Small interfering RNAs (siRNAs) are small (around 20 nucleotides), noncoding RNAs endowed with the ability to downregulate gene expression through a process known as RNA interference (RNAi) [175]. This technology involves a post-transcriptional mechanism that inhibits gene expression by promoting the cleavage on a specific region of the target messenger RNA (mRNA) and shows promising therapeutic results in cancer treatment.

However, the clinical use of siRNA is still limited by its rapid degradation, nonspecific distribution, and poor cellular uptake [176]. Therefore, delivery systems able to preserve the integrity and improve the delivery of siRNAs to the site of action in the cytosol are required to develop successfully siRNA-based therapeutics.

**Figure 5:**
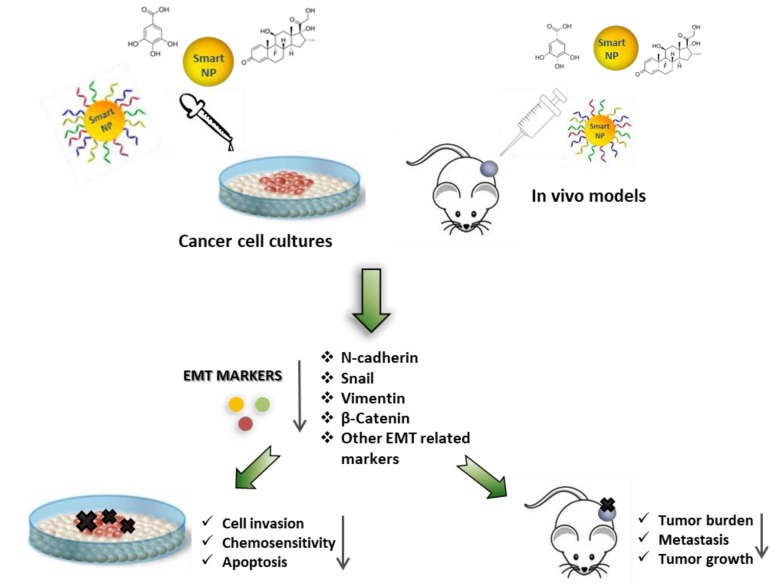
Nanoparticles as a tool for the delivery of natural substances, synthetic therapeutics, and nucleic acids to revert the EMT phenotype. Schematic representation showing the effects of nanoparticles functionalized with natural substances, synthetic therapeutics, and nucleic acids, in targeting the EMT program. The resulting EMT reversion (MET) is able to limit tumor cell migration and invasion and to enhance chemosensitivity, as well as to reduce tumor growth.

Recently, some nanomaterials have been exploited to deliver therapeutic siRNAs against key EMT-related genes in cancer cells, which are commented on below and resumed in Figure 5.

#### 4.4.2. SNAIL-1

SNAIL-1 is the most characterized EMT TF, originally discovered as an E-cadherin repressor [177]. The involvement of SNAIL-1 in the regulation of EMT and metastasis and its roles in both drug and immune resistance [29] make it an attractive target for novel anticancer drugs. 

Chitosan is a biocompatible and biodegradable polymer used in nanomedicine for siRNA delivery [178]. Some studies have exploited carboxymethyl dextran (CMD) chitosan nanoparticles (ChNPs) for the double encapsulation of Snail siRNA and anticancer drugs such as doxorubicin (DOX) and SN38, for synergistic cancer therapy [79,80,81]. These nanocomplexes led to EMT inhibition and significant changes in the expression of EMT-related genes. The reversion of the mesenchymal phenotype was accompanied to apoptotic cell death, inhibition of cell growth and metastasis, and enhancement in drug sensitivity in colorectal, lung, and prostate cancer and in vivo [79,80,81]. 

#### 4.4.3. ZEB1

The EMT TF ZEB1 promotes carcinogenesis and metastasis by inducing an invasive phenotype and by conferring stem-like properties [27]. Therefore, targeting this TF may reduce cancer malignancy and improve the oncologic outcome for cancer patients.

Multifunctional polypeptide micelle nanoparticles (NPs) have been employed as nanocarriers for the delivery of ZEB1-specific siRNA in combination with DOX therapy in lung cancer [82]. The combined treatment synergistically reduced EMT, repressed CSC proprieties in vitro and in vivo, and finally suppressed metastasis [82]. Therefore, targeting ZEB1 with nanostructures represents a promising strategy to inhibit EMT and cancer metastasis.

#### 4.4.4. TWIST1

Differently than SNAIL-1 and ZEB1, TWIST1 is a more potent mesenchymal inducer than epithelial repressor [179]. TWIST1 plays a critical role in some physiological processes leading to cancer metastasis [180] and has recently gained significant interest in cancer therapy.

A plethora of studies reported that siRNAs against TWIST1 delivered by nanoparticles inhibited the EMT process in several tumors, leading to the reduction of tumor progression and metastasis and sensitizing cancer cells to chemotherapy drugs and radiotherapy [83,84,85,86,87,111,112,181,182].

In this regard, Finlay and colleagues reported that the delivery of TWIST1-targeting siRNAs with a polyethyleneimine-coated mesoporous silica nanoparticle (MSN) was able to revert the mesenchymal phenotype of MDA-MB-435S melanoma cells, thus leading to the inhibition of migratory potential and tumor burden in vivo [85].

In another study, a TWIST1-specific siRNA was delivered in ovarian and breast cancer cells by polyamidoamine dendrimers (PAMAM) and hyaluronic acid conjugated mesoporous silica nanoparticles (MSN-HAs). These nanoformulations inhibited the EMT program, reversing chemoresistance to cisplatin and leading a reduction of tumor growth in mice models [83,84,87].

Li and coworkers reported that pH-sensitive polymeric nanomaterials loaded with siRNAs targeting TWIST1 in combination with paclitaxel suppressed EMT, reducing tumor growth and metastasis as well as ECM degradation in breast cancer cells and orthotopic mice models [111,112].

#### 4.4.5. Nogo-B Receptor

Nogo-B receptor (NgBR) plays a fundamental role in regulating EMT and promoting chemoresistance and metastasis in breast and lung cancer cells [183,184,185].

In a recent study, a polymeric nanostructure responsive to the tumor extracellular acidity has been developed for the delivery of siRNAs against NgBR in tumor tissues [88]. The nanoparticles presented a negative surface charge in blood circulation and healthy tissues, but the charge changed to positive through the hydrolysis of a sensitive molecule exposed on the surface of the micelles. This change promoted the cellular uptake of siRNAs, leading the inhibition of NgBR in tumor-bearing tissues.

Thus, the system efficiently inhibited the EMT process resulting in attenuated cell migration and distant metastasis in breast cancer [88].

#### 4.4.6. β3 Integrin

Among integrin isoforms, adhesion receptors implicated in cell-to-cell and cell-ECM interactions, β3 integrin has been mainly studied in tumors due to its effect on angiogenesis [186]. To this respect, several nanomaterials have been used to target β3 integrin in vivo resulting in the blockage of mesenchymal phenotype and cancer metastasis [89,90]. For instance, the multifunctional cationic lipid-based carrier (1-aminoethyl)iminobis[N-oleicylsteinyl-1-aminoethyl)propionamide] (ECO) was used to deliver siRNAs targeting β3 integrin in triple-negative breast cancer (TNBC) cells. The treatment with ECO/siRNAs nanostructures resulted in the inhibition of cellular migration, tumor spheroid formation, and 3-dimensional organoid growth [89,90]. Moreover, ECO lipid nanocomplexes led to a reduction of tumor growth and metastasis in vivo [89,90], thus representing a promising therapeutic approach to modulate signaling pathways EMT-related involved in TNBC.

#### 4.4.7. DCAMKL-1

The novel putative intestinal and pancreatic stem cell marker DCAMKL-1 [187,188], a microtubule-associated kinase, plays a key role in tumorigenesis by downregulating tumor suppressor microRNAs thus inducing tumor promoters specific targets such as ZEB1, ZEB2, and Notch-1 [189,190].

Polymeric nanostructures have been employed to deliver DCAMKL-1 siRNA to modulate potential essential genes cancer regulators [91]. It has been reported that the administration of these nanocomplexes into HCT116 xenograft mice models resulted in the upregulation of EMT inhibitor miR-200a and downregulation of EMT-associated markers leading tumor growth arrest via let-7a and miR-144 dependent mechanisms [91]. Hence, nanoparticle-based delivery of siRNAs targeting DCAMKL-1 might offer a novel strategy for cancer treatment through the regulation of oncogenic signaling.

#### 4.4.8. Wnt-1

Wnt-1 is an extracellular protein implicated in morphogenesis. In tumors, Wnt-1 is frequently upregulated, and its activity is linked to the stabilization and activation of the oncogene β-catenin, which leads to EMT activation [17]. Hence, dysregulation in Wnt-1/β-catenin pathway plays a critical role in carcinogenesis and chemoresistance and represents a valid target for novel cancer therapy [191]. Furthermore, Wnt-1 can be targeted to increase the efficacy of complementary therapies. In this regard, polyethylene glycol-polyethyleneimine-chlorin e6 (PEG-PEI-Ce6) nanoparticles were used to deliver siRNAs against Wnt-1 in an oral squamous cell carcinoma that was treated with photodynamic therapy (PDT). In this case, it was observed that PDT alone led to an activation of the EMT process, inhibiting the antitumoral effect. However, the combination strategy mentioned above produced a significant reduction of some proteins crucial to the EMT process, enhancing the antitumoral activity of PDT [92].

#### 4.4.9. STAT3

STAT3 is a transcription factor activated by cytokines elicited during inflammatory–immune responses such as interleukin (IL)-6 and -10. STAT3 plays a role in EMT by cooperating with TGFβ signaling [192]. Constitutive activation of STAT3 has been linked to cell growth, differentiation, survival, and metastasis [193,194]. Among the different drugs targeting STAT3, some nanomedicines have been exploited for this aim and represent a promising therapeutic approach.

In this sense, cationic solid lipid nanoparticles (SLN) were employed to deliver decoy oligodeoxynucleotides (ODN) to block STAT3 in ovarian cancer. SLN-STAT3 decoy ODN complexes led the reversion of EMT phenotype, reduced cell invasion, and increased apoptotic and autophagic cell death [93].

#### 4.4.10. AXL Kinase

AXL is a member of the receptor tyrosine kinase (RTK) family and can enable several downstream pathways through interaction with Epidermal growth factor receptor (EGFR) [195].

Additionally, a recent study showed that the overexpression and activation of AXL induced the EMT program in cancer cells and was associated with increased metastasis and chemoresistance [196].

For these reasons, AXL kinase represents a valid therapeutic target to counteract cancer. In this sense, Suresh et al. developed antibody functionalized gelatin nanoparticles (GAb) for effective cytoplasmatic delivery of anti-AXL siRNA in lung cancer cells [94]. These nanostructures repressed EMT by downregulating MMPs production and key EMT-related proteins, such as N-cadherin and vimentin, sensitizing H820 and H1975 cell lines to tyrosine kinase inhibitors [94].

### 4.5. Delivery of miRNAs Inhibiting EMT

miRNAs are endogenous, small, noncoding RNAs that function in the regulation of gene expression, whose dysregulation is related to many pathological processes, including EMT and tumorigenesis [197]. Hence, re-establishing endogenous intracellular levels of miRNAs may reverse EMT and counteract cancer progression. However, the low stability and the difficulty in delivering miRNAs into cells limits its application [198]. For these reasons, nanocarriers are explored to deliver miRNA derivatives to block oncogenic miRNAs or to restore the physiological levels of tumor suppressor miRNAs. Selected miRNAs involved in the regulation of the EMT delivered by nanomaterials are commented below.

#### 4.5.1. miRNA-21

miRNA-21 has been shown to play a pivotal role in cancer initiation and progression in many tumors and represents a target for the treatment of pancreatic cancer [199]. In a recent study, a multifunctional nanoscaffold was developed using polyethylene glycol–polyethylenimine–magnetic iron oxide NPs (PEG-PEI-IONPs) for combined therapy of miRNA-21 antisense oligonucleotides (ASO-miR-21) and gemcitabine (Gem) [95]. This nanocomplex suppressed EMT, leading to the reduction of the migration and invasion capabilities of pancreatic cancer cells in vitro and the tumor growth and metastasis in vivo [95]. 

#### 4.5.2. miRNA-139

miRNA-139 has been found to be downregulated in various types of cancer, including colorectal cancer [200,201]. Further data have shown that low miR-139 expression is related to the aggressiveness of colon cancer [201] and a high incidence of chemoresistance and metastasis via EMT activation [200]. Therefore, miR-139 might represent a therapeutic target by counteracting EMT signaling. In this sense, multifunctional lipid–polymer hybrid nanoparticles (LPNs) [202] composed of PLGA and PEG-lipids has been used to deliver miR-139 in colorectal cancer cells [96]. This nanostructure was modified with two cell-penetrating peptides (CPP) to enhance targeting and penetrating characteristics in colorectal cancer cells. The system was able to suppress EMT and cancer cell migration by downregulating β-catenin and Slug, enhancing the sensitivity to afatinib [202] concomitantly.

#### 4.5.3. miRNA-200c

miRNA-200c, a member of the miRNA-200 family, can reverse the EMT by attenuating the invasive ability of epithelial cells and increasing E-cadherin expression [203,204,205]. Liu and coworkers developed gelatinases stimuli nanoparticles in order to co-deliver miRNA-200c and docetaxel (DOC). These nanomaterials inhibited EMT in both CSCs and non-CSCs gastric cancer cells [97] and significantly enhanced the cytotoxicity of DOC. Besides, this nanoformulation suppressed tumor growth in vivo, representing a promising strategy for co-delivery of nucleic acid and drugs, and at the same time, to suppress CSCs and non-CSC cancer cells.

#### 4.5.4. Delivery of miRNAs Mimics

AuNPs were employed as a nanocarrier for the delivery of microRNA mimics to restore a physiological miRNAs signature in uveal melanoma [206]. In particular, this strategy was used to increase the levels of two tumor suppressor miRNAs, miRNA-34a and miRNA-144, which play a crucial role in EMT inhibition [207,208]. Hence, restoring a standard miRNAs profile using AuNPs led to chemosensitivity and cell death [206]. 

## 5. NP-Mediated Toxicology by Modulating EMT

Despite the potential advantages of the described nanostructures, it is necessary to remember that the use of these nanomedicines may display some toxicity. Interestingly, both the toxicity and the therapeutic effect observed may derive from the modulation of the EMT process (Table 3).

It has been reported that BEAS-2B human bronchial epithelial cells under chronic exposure to silver nanoparticles undergo an increase of anchorage-independent cell growth, collagen deposition, cell migration, and invasion [209,210]. Also, an antiapoptotic effect and an induction of EMT pathways were observed, indicating that BEAS-2B cells chronically exposed to low dose of AgNPs could acquire some features characteristic of tumorigenic cells [209,210].

Increasing reports showed that treatment with silica nanoparticles (SiNPs) might lead to inflammation, which represents an important co-cause in tumor onset [219]. A recent report demonstrated that BEAS-2B cells co-cultured with human mononuclear (THP-1) cells when treated with benzo[a]pyrene-7, 8-dihydrodiol-9, 10-epoxide (BPDE) and spherical SiNPs showed activation of EMT and tumorigenic features [211]. Interestingly, co-treatment of SiNPs and BPDE promoted EMT of BEAS-2B cells via the AKT pathway by inducing the release of SDF-1 from THP-1 cells [211]. Although direct carcinogenesis by SiNPs treatment has not been proven so far, this evidence suggests that SiNPs can promote tumor development when combined with BPDE and assume relevance for explaining the high incidence of lung cancer upon environmental exposure.

Several studies suggest that cerium oxide nanoparticles (CeO_2_-NPs) from diesel engines represent a serious health concern since long-term exposure to these nanocompounds may lead to inflammation and lung fibrosis [220,221].

CeO_2_-NPs-induced lung fibrosis has been shown to be linked with the accumulation of these nanomaterials in the lungs, the induction of inflammatory and fibrotic cytokines, for example, TGFβ1, and the dysregulation of the mediators playing a pivotal role in ECM remodeling [220].

Recently, it was reported that CeO_2_-NPs exposure can induce lung fibroblast activity and induce EMT in alveolar type II (ATII) cells isolated from CeO_2_-NPs-exposed rats playing a pivotal role in lung fibrosis [212].

Carbon nanotubes (CNTs) are of great interest due to their peculiar chemical and physical properties and their potential applications in engineering, industry, and medicine. However, with the increase of CNT production and utilization, the adverse health effects of chronic exposure to these nanomaterials have emerged. Particularly, chronic inflammation, fibrosis, and carcinogenesis have been observed in human lung epithelial cells after long-term exposure to CNTs [222,223,224].

Interestingly, several studies report that exposure to CNTs can induce EMT and fibrosis, leading to oncogenic transformation, tumor progression, and metastasis in bronchial, mesothelial, and lung epithelial cells [213,214,215,216,217].

Although the toxicity of the majority of nanomaterials is related mainly to the respiratory system, toxicity towards other tissues was also reported.

For instance, chronic exposure to TiO_2_-NPs led to substantial renal injury in mouse kidney [225], which was attributed to chronic renal inflammation and fibrosis [226].

More recent studies linked this fibrotic damage to the activation of Wnt pathway and EMT induction [218].

These and other studies [227,228,229], indicate that EMT modulation mediated by certain nanomaterials can potentially represent a risk for the respiratory system. However, the involvement and nature of EMT activation in the pathogenesis of the above-described diseases need further investigation.

## 6. Conclusions

EMT induction leads to an extensive reprogramming of the cell proteome implicated in many physio-pathological conditions, including tumor transformation and metastasis.

There are many pieces of evidence suggesting that targeting EMT may be exploited as a therapeutic strategy itself, or to enhance the efficacy of chemotherapy drugs [3,4,5]. In this regard, nanostructures can be employed to modulate EMT or to deliver therapeutic molecules against EMT-related pathways. 

Thus, the application of these functional nanomaterials results in apoptosis induction, inhibition of cell invasion and migration, and increased chemosensitivity and therapeutic efficacy. 

Despite these benefits reported, it should be kept in mind that, in some cases, nanomaterials may overstimulate EMT in healthy tissues leading to dangerous effects, including inflammation, fibrosis, and neoplastic transformation.

In conclusion, the findings summarized in this review suggest that EMT inhibition with nanoparticle-based strategies may interfere with various steps of tumor transformation, providing an effective therapeutic approach against cancer.

## Figures and Tables

**Table 1:** Nanoparticles (NP)-mediated EMT modulation in cancer cells.

Entry	NPs	Combined Treatments	Target Cells	Cancer Tissues	Regulation of EMT Markers	Biological Effects	Refs
1	Gold nanoparticles	none	A2780, OVCAR5 SKOV3-ip	Epithelial ovarian cancer	E-cadherin↑ N-cadherin↓ Snail↓ p42/44↓ MAPK↓	Inhibition of cell proliferation; Reversion of epithelial plasticity; Inhibition of EMT	[48]
2	Gold nanoparticles	none	A2780, OVCAR5 and SKOV3-ip	Epithelial ovarian cancer	ALDH1↓, CD44↓, CD133↓, Sox2↓, MDR1↓, ABCG2↓ Akt signaling↓ NF-κB signaling↓ E-Cadherin↑ β-Catenin↑ Vimentin↓ α-SMA↓	Sensitivity to cisplatin; Suppression cancer stem cell proprieties; Inhibition of EMT	[49]
3	Gold nanoparticles	none	PANC-1, AsPC-1 and HPAF II	Pancreatic cancer	E-cadherin↑ N-cadherin↓ Vimentin↓	Sensitivity to gemcitabine; Suppression cancer stem cell proprieties; Inhibition of EMT	[50]
4	Gold nanoparticles	none	HUVECs B16F10	Melanoma Blood vessels	E-Cadherin↑ ZO-1↑ Vimentin↓ C-myc↓ MMP2↓	Inhibition of cell migration; Inhibition of EMT	[51]
5	Gold nanorods	none	HeLA MCF-7	Cervical cancer Breast cancer	Vimentin↓ N-cadherin↓	Inhibition of collective migration; Decrease of EMT markers	[52]
6	Gold Nanoparticles	Cold plasma	T98G A459	Glioblastoma Lung cancer	E-Cadherin↑ N-Cadherin↓ Slug↓ ZEB1↓ PI3K/AKT patwhay ↓	Apoptosis; Reduction of cell proliferation; Inhibition of EMT; Decrease in sphere formation; Decrease in self-renewal capacity	[53]
7	Titanium dioxide	none	A459	Epithelial lung cancer	Smad2/3↓ E-Cadherin↑ N-cadherin↓	Inhibition of TGF-β-Mediated Cell Migration; Suppression of TGF-β-Induced EMT; Attenuation of TGF-β Signaling	[54]
8	Titanium dioxide Silicon dioxide	none	LX-2	Fibrosis	N-Cadherin↓ E-Cadherin↑	Inhibition of EMT; Inhibition of fibrosis; Reduction of adhesion and migration profiles	[55]
9	ZnO Nanostructures	none	T98G SNU-80 H-460	Glioblastoma Thyroid cancer Lung cancer	N-Cadherin↓ ZEB1↓	Cell death; Apoptosis; Reduction of cell invasion; Inhibition of EMT	[56]
10	D, L-lactic-co-glycolic acid (PLGA)	α–Mangostin	PANC-1, AsPC-1, MIA PaCa-2; Human CSCs, Kras^G12D^ mouse CSCs	Pancreatic cancer	E-cadherin↑ N-cadherin↓ Slug, Snail1, ZEB1↓ Nanog, c-Myc, Oct4↓ Shh pathway↓ Gli targets↓	Inhibition of cancer growth; development; metastasis; inhibition of pluripotency; Inhibition of EMT	[57]
11	D, L-lactic-co-glycolic acid (PLGA)	Anthothecol	PANC-1, AsPC-1, MIA PaCa-2; Human CSCs, KrasG12D mouse CSCs	Pancreatic cancer	E-cadherin↑ N-cadherin↓ Slug, Snail, ZEB1↓ Nanog, c-Myc, Oct4↓ Shh pathway↓ Gli targets↓	Inhibition of cell proliferation; invasion; migration; induction of apoptosis; inhibition of pluripotency; Inhibition of EMT	[58]
12	D, L-lactic-co-glycolic acid (PLGA	Wedelolactone	MDA-MB-231 Breast cancer stem cells	Triple negative breast cancer	E-Cadherin↑ N-Cadherin↓ TWIST1↓ Snail↓ Vimentin↓	Retarded migration and invasion; Reduction of cell viability; Apoptosis; Inhibition of EMT; Reduction of pluripotency; Drug sensitivity to paclitaxel	[59]
13	D, L-lactic-co-glycolic acid (PLGA)	Salinomycin	AsPC-1	Pancreatic cancer	E-Cadherin↑ β catenin↑ TGFβ R-1↓ TGFβ R-2↓	Inhibition of EMT; Apoptosis	[60]
14	Polymeric micelles	Salinomycin	A459	Lung cancer	Vimentin↓	Inhbition of EMT; Reversion to epithelial phenotype; Reduction of cell migration; Prevention of P-gp efflux	[61]
15	Silver nanoparticles	Gallic Acid	A459	Lung cancer	Vimentin↓ N-cadherin↓ Snail1↓ E-cadherin↑	Loss of radiation-induced metastasis; Inhibition of EMT	[62]
16	Curcumin loaded selenium nanoparticles (Se-Cu NPs)	Curcumin	HCT116	Colon cancer	CD44↓ N-Cadherin↓	Induction of autophagy; Induction of apoptosis; Induction of cell cycle arrest; Inhibition of EMT	[63]
17	Curcumin loaded selenium nanoparticles (Se-Cu NPs); CD44-targeted DOX loaded nanoparticles (PSHA-DOXNPs)	Curcumin, Doxorubicin	HCT116	Colon cancer	N-Cadherin↓ Vimentin↓ Snail1↓ CD44↓ MMP2↓ MMP4↓	Induction ROS levels; Decreased mitochondrial membrane potential; Induction cell cycle arrest; Apoptosis; Inhibition of EMT	[64]
18	Gold Nanoparticles	Quercetin	MCF-7 MDA-MB-231 HUVECs	Breast cancer	E-Cadherin↑ N-Cadherin↓ Vimentin↓ Snail↓ Slug↓ TWIST1↓ MMP2/9↓ EGFR/VEGFR-2 signalling↓	Inhibition of EMT; Inhibition of angiogenesis; Inhibition of cell invasion	[65]
19	Liposomal	Quercetin	Eca109/9706	Esophageal squamous cell carcinoma	E-Cadherin↑	Apoptosis; Inhibition of EMT	[66]
20	Mesoporous silica; PEG-PLA micelles	Epigallocatechin gallate/iron	4T1	Mouse breast cancer	MMP2/9 ↓ VEGF↓ Vimentin↓ E-cadherin↑	Suppression of metastasis; Inhibition of EMT	[67]
21	Layered double hydroxide	Etoposide	U87MG Glioblastoma stem cells (GSCs)	Glioblastoma	Sox2↓ Oct4↓ Nanog↓ Nestin↓ Snail↓ N-Cadherin↓ E-Cadherin↑ PI3K/AKT/mTOR↓ WNT/GSK3β/β-catenin↑	Inhibition of cell proliferation; Down-regulation of GSCs stemness; Inhbition of EMT	[68]
22	Liposomes	ADH-1 peptide Paclitaxel	MCF7-paclitaxel resistant	Breast cancer	N-Cadherin↓ E-Cadherin↑	Improvement of chemosensitivity; Inhibition of cell migration; Inhibition of EMT	[69]
23	Liposomes	ADH-1 peptide DOX Hyaluronic Acid	A459	Lung cancer	N-Cadherin↓ CD44↓	Drug sensitivity; Reduction of cell migration; Inhibition of EMT	[70,71]
24	Gold nanoparticles	Dexamethasone (DSH) thiol derivative Withaferin (WFA)	B16F10	Murine melanoma	E-Cadherin↑ Vimentin↓ pAKT/AKT signalling↓	Induction of apoptosis; Inhibition of cell cycle; Induction of MET; Inhibition of EMT	[72]
25	Zinc arsenite	Arsenic trioxide	Hep3b, HepG2, Bel7402 and MHCC97L	Liver cancer	E-Cadherin↑ Vimentin↓ Slug↓ SHP-1/JAK2/STAT3↓	Suppress tumor initiation and growth; Suppression metastasis Inhibition stemness and EMT	[73]
26	Albumin based nanoparticles	Arsenic trioxide	in 5-8F CNE-2	Nasopharyngeal carcinoma	E-Cadherin↑ N-Cadherin↓ Vimentin↓	Inhibition of colony formation; Inhibition of cell invasion; Inhibition of EMT	[74]
27	Liposome	^188^Re	ES-2-luc	Ovarian cancer	E-Cadherin↑ Vimentin↓ p53↑	Switch to mitochondrial phosphorylation; Reactivation of p53 function; Inhibition of EMT	[75]
28	Liposome	^188^Re	FaDu	Head and neck squamous cell carcinoma	Let-7↑	Suppression of tumor growth	[76]
29	Liposome	^188^Re	FaDu, SAS	Head and neck squamous cell carcinoma	E-Cadherin↑ N-Cadherin↓ TWIST1/2 ↓ Vimentin↓ ZEB1↓ Slugs↓	Inhibition of cell proliferation; Cell death; Inhibition of EMT	[77]
30	Liposome	Simvastatin, Paxicitel	A549T PC9 TAM (tumor associated macrophages)	Lung and prostate cancer	FAK↓ ERK/AKT↓ TNF-α↑ TGFβ↓ LXR/ABCA1↓ E-Cadherin↑ Vimentin↓	Inhibition of EMT; Sensitization to paxicitel; Repolarization of TAM; Regulation of cholesterol metabolism	[78]
31	Carboxymethyl dextran (CMD)-chitosan nanoparticles (ChNPs)	Snail siRNA DOX	HCT-116	Colon cancer	MMP9 ↓ Vimentin↓ E-cadherin↑	Inhibition cell growth; apoptosis; inhibition of migration; Inhibition of EMT	[79]
32	Carboxymethyl dextran (CMD)-chitosan nanoparticles (ChNPs)	Snail siRNA SN38	PC-3	Prostate cancer	E-cadherin↑ Claudin-1↑	Reduction of cell proliferation; Reduction of cell migration; Inhibition of EMT	[80]
33	Carboxymethyl dextran (CMD)-chitosan nanoparticles (ChNPs)	Snail siRNA HMGA2 siRNA DOX	A459	Lung cancer	HMGA2↓ E-cadherin↑ Vimentin↓ MMP9↓	Apoptosis; Reduction in cell migration; Drug sensitivity; Inhibition of EMT	[81]
34	Polypeptide micelles (PEG–PLL–PLLeu)	ZEB1 siRNA DOX	H460	Non-small cell lung cancer (NSCLC)	ZEB1↓ E-cadherin↑ SOX2↓ ABCG2↓	Inhibition of EMT; Repression of CSC properties; Reduction of cell invasion; Sensitivity to DOX	[82]
35	Polyamidoamine dendrimers (PAMAM) and Hyaluronic-acid conjugated mesoporous silica nanoparticles (MSN-Has)	TWIST1 siRNA Cisplatin	F2 Ovcar8	Ovarian cancer	Vimentin↓ E-Cadherin↑ N-Cadherin↓	Chemosensitivity to cisplatin; Inhibition of EMT	[83,84]
36	Mesoporous Silica	TWIST1 siRNA	MDA-MB-435S	Melanoma	Vimentin↓ CCL2↓	Inhibition of migration; Inhibition of EMT	[85]
37	Chitosan-coated nanoparticles	TWIST1 siRNA	CNE2	Nasopharyngeal carcinoma	p-ERK↑	Sensitivity to radiation; Irradiation-induced apoptosis	[86]
38	Polyamidoamine dendrimers (PAMAM)	TWIST1 siRNA	SUM1315	Triple negative breast cancer	N-Cadherin↓ Vimentin↓	Reduction of cell migration and invasion; Inhibition of EMT	[87]
39	(PLGA)2-PEI-DMMA nanoparticles	NgBR siRNA	HUVECs MDA-MB-231 4T1	Breast cancer	Vimentin↓ E-Cadherin↑	Inhibition of endothelial cell migration; Suppression of cancer cell invasion Normalization of tumor blood vessel; Inhibition of EMT	[88]
40	ECO lipid carrier	β3 integrin siRNA	MDA-MB-231	Triple negative breast cancer	PAI-1↓ N-cadherin↓ E-cadherin↑ CK19↑	Inhibition of TGFβ-mediated cytostasis; Inhibition of TGFβ-mediated EMT; Inhibition of TGFβ-mediated invasion; Inhibition of 3-dimensional organoid growth; Inhibition of EMT	[89]
41	ECO lipid carrier	DANCR siRNA	MDA-MB-231 BT549	Triple negative breast cancer	β-catenin↓ ZEB1↓ Stat proteins↓ N-cadherin↓ Survivin↓ WNT signaling↓	Inhibition of cell invasion; Inhibition of cell migration; Reduction of survival; Reduction in tumor spheroid Formation; Inhibition of cell proliferation Inhibition of EMT	[90]
42	Poly(lactide-co-glycolide) acid nanoparticles (PLGA NPs)	DCAMKL-1 siRNA	HCT116	Colon cancer	miRNA 200a↑ miRNA let-7a↑ E-Cadherin↑ ZEB1/2↓ Snail↓ Slug↓	Inhibition of tumor growth; Inhibition of EMT	[91]
43	Polyethylene glycol-polyethyleneimine-chlorin e6	Wnt-1 siRNA	KB	Oral squamous cell carcinoma	Wnt-1 ↓ β-catenin↓ Vimentin↓	Inhibition of cell growth; sensibility to PDT; Inhibition of EMT	[92]
44	Cationic solid lipid nanoparticles (SLN)	STAT3 decoy oligodeoxynucleotide	A2780 SKOV3	Ovarian cancer	E-Cadherin↑ Snail↓ MMP9↓	Induction of cell death; Apoptotic and autophagy cell death; Inhibition of invasion; Inhibition of EMT	[93]
45	Gelatin nanoparticles	AXL siRNA	H820 and H1975 erlotinib-resistant	Non-small cell lung cancer	MMP9↓ MMP2↓ Vimentin↓ N-cadherin↓	Overcome of chemoresistance to tyrosine kinase inhitors; Inhbition of EMT	[94]
46	Polyethylene glycol–polyethylenimine–magnetic iron oxide (PEG-PEI-IONPs)	microRNA-21 antisense oligonucleotides; Gemcitabine	PANC-1 MIA PACA-2	Pancreatic cancer	PTEN↑ PDCD4↑ Vimentin↓ E-Cadherin↑	Inhibition of cell proliferation; Apoptosis; Inhibition of invasion and cell migration; Inhibition of EMT	[95]
47	Lipid–polymer hybrid nanoparticles modified with CPP	Afatinib; miR-139	Caco-2	Colorectal cancer	E-Cadherin↑ β-catenin↓ Slug↓	Inhbition of EMT; Sensitivity to afatinib; Reduced cancer cell migration	[96]
48	Gelatinases-stimuli poly(ethylene glycol)-peptide-poly(ε-caprolactone) copolymer	miR-200c Docetaxel	BGC-823 CSCs and non-CSCs	Gastric adenocarcinoma	E-Cadherin↑ CD44↓	Drug sensitivity to DOC; Inhibition of EMT	[97]

**Table 2:** In vivo assays performed in the studies reported in the review.

Entry	NPs	Compound/Drugs Carried	Animal Models	Diseases Model	Biological Effects	Refs
1	Gold	none	orthotopic ovarian tumor models (A2780 cells or SKOV3-ip)	Ovarian cancer	Inhibition of tumor growth; Inhibition of metastasis; Inhibition of EMT	[48]
2	Gold	none	Female athymic nude mice orthotopic ovarian tumor models (SKOV3-ip)	Ovarian cancer	Drug sensitivity to cisplatin; Reduction of tumor growth; Inhibition of EMT	[49]
3	Gold	none	Female C57BL6 mice	Melanoma	Improvement of vascular morphology; Increase vascular perfusion and decrease permeability; Alleviation of hypoxia; Reduction of lung metastasis; Inhibition of EMT	[51]
4	Gold Nanoparticles	Cold plasma	Athymic balb/c female nude mice U87MG xenograft model	Glioblastoma Lung cancer	Reduction tumor growth	[53]
5	Titanium dioxide	none	Wild-type zebrafish AB strain; Male C57BL/6 mice	Caudal regeneration; Colitis	Impairment of Caudal Fin Regeneration; Colon injury	[54]
6	D, L-lactic-co-glycolic acid (PLGA)	α–Mangostin	KC (Pdx^Cre^;LSL-Kras^G12D^); KPC (Pdx^Cre^;LSLKras^G12D^;LSL-Trp53^R172H^	Pancreatic cancer	Inhibition of cancer growth; development; metastasis; Inhibition of EMT	[57]
7	D, L-lactic-co-glycolic acid (PLGA)	Wedelolactone	Female Swiss Albino mice	Triple negative breast cancer	Reduction tumor volume; Reduction cancer stem cells	[59]
8	D, L-lactic-co-glycolic acid (PLGA)	Salinomycin	Female athymic nude mice AsPC-1-luc orthotopic model	Pancreatic cancer	Reduction tumor growth; Inhibition of EMT	[60]
9	Curcumin loaded selenium nanoparticles (Se-Cu NPs)	Curcumin	Ehrlich’s ascites carcinoma (EAC)-bearing mice.	Colon cancer	Reduction tumor mass; Increasing mean survival time	[63]
10	Curcumin loaded selenium nanoparticles (Se-Cu NPs); CD44-targeted DOX loaded nanoparticles (PSHA-DOXNPs)	Curcumin, Doxorubicin	Ehrlich’s ascites carcinoma (EAC)-bearing mice.	Colon cancer	Decreased tumor burden; Increased survival	[64]
11	Gold Nanoparticles	Quercetin	Female Sprague-Dawley rats	Breast cancer	Inhibition of tumor growth; Inhibition of metastasis	[65]
12	Mesoporous silica; PEG-PLA micelles	Epigallocatechin gallate/iron	Female balb/c mice	Breast cancer	Suppression of metastasis; Inhibition of EMT	[67]
13	Layered double hydroxide	Etoposide	Female BALB/c nude mice	Glioblastoma	Reduction of tumor growth; Induction of apoptosis; Inhibition of pluripotency; Inhibition of EMT	[68]
14	ADH-1 peptide-modified liposomes	Paclitaxel	Female BALB/c nude mice	Breast cancer	Inhibition of tumor growth; Enhancement of chemosensitivity	[69]
15	Gold nanoparticles	Dexamethasone (DSH) thiol derivative Withaferin (WFA)	Old female C57BL/6J mice	Melanoma	Induction of apoptosis; Inhibition of tumor growth; Reduction in mice mortality; Inhibition of EMT	[72]
16	Albumin based nanoparticles	Arsenic trioxide	Xenograft model	Nasopharyngeal carcinoma	Reduction tumor growth; Inhibition of EMT	[74]
17	Liposome	^188^Re	Balb/C nude mice Orthotopic xenograft model	Ovarian cancer	Reactivation p53; Switch to oxidative phosphorylation; Inhibition of EMT	[75]
18	Liposome	^188^Re	Balb/C nude mice Orthotopic xenograft model	Head and neck squamous cell carcinoma	Suppression of tumor growth	[76]
19	Liposome	^188^Re	Balb/C nude mice Orthotopic xenograft model	Head and neck squamous cell carcinoma	Suppression of tumor growth; Survival; Inhibition of EMT; Inhibition of proliferation markers; Accumulation in tumor site	[77]
20	Liposome	Simvastatin, Paxicitel	A549T xenograft mouse model	Lung cancer	Reversion of chemoresistance to PTX in vivo; Arrest of cell growth; Inhibition of EMT; Change of cell polarization of TAM in vivo	[78]
21	Polypeptide micelles (PEG–PLL–PLLeu)	ZEB1 siRNA DOX	Female BALB/c nude mice H460 xenograft model	Non-small cell lung cancer (NSCLC)	Inhibition of EMT; Repression of CSC properties; Inhibition of metastasis; Inhibition of tumor growth	[82]
22	Polyamidoamine dendrimers (PAMAM) and Hyaluronic-acid conjugated mesoporous silica nanoparticles (MSN-Has)	TWIST1 siRNA Cisplatin	NOD.Cg-Prkdc^scid^Il2rg^tm1Wjl^/SzJ (NSG) mice	Ovarian cancer	Inhibition of tumor growth; Sensitivity to cisplatin; Inhibition of EMT	[83,84]
23	Mesoporous Silica	TWIST1 siRNA	NOD.Cg-Prkdc^scid^ Il2rg^tm1Wjl^/SzJ (NSG)	Melanoma	Reduction of tumor burder; Inhibition of EMT	[85]
24	PEI-PDHA PEG-PDHA P85-PEI/TPGS	TWIST1 siRNA Snail siRNA Paclitaxel	4T1 tumor-bearing mice models	Breast cancer	Reduction of metastasis; Inhibition of cell invasion; Inhibition of ECM degradation Inhibition of tumor growth	[111,112]
25	Polyamidoamine dendrimers (PAMAM)	TWIST1 siRNA	NOD.Cg-PrkdcscidIl2rgtm1Wjl/SzJ (NSG) mice	Breast cancer	Biodistribution in tumor site	[87]
26	(PLGA)2-PEI-DMMA nanoparticles	NgBR siRNA	Female BALB/c nude mice Orthotopic model	Breast cancer	Reduction of metastasis	[88]
27	ECO lipid carrier	β3 integrin	Female nude mice (nu/nu Balb/c background)	Triple negative breast cancer	Reduction of primary tumor burden; Inhibition of metastasis; Inhibition of EMT	[89]
28	ECO lipid carrier	DANCR siRNA	Nude athymic mice MDA-MB-231 and BT549 xenograft model	Triple negative breast cancer	Reduction of tumor growth; Inhibition of EMT	[90]
29	Poly(lactide-co-glycolide) acid nanoparticles (PLGA NPs)	DCAMKL-1 siRNA	Male athymic nude mice (NCr-nu/nu) HCT116 cells orthotopic models	Colon cancer	Arrest in tumor growth; Inhibition of EMT; Regulation of oncogenic miRNAs network	[91]
30	Polyethylene glycol–polyethylenimine–magnetic iron oxide (PEG-PEI-IONPs)	microRNA-21 antisense oligonucleotides; Gemcitabine	Female BALB/c nude mice	Pancreatic cancer	Reduction tumor growth; Inhibition of metastasis	[95]
31	Gelatinases-stimuli poly(ethylene glycol)-peptide-poly(ε-caprolactone) copolymer	miR-200c Docetaxel	BGC-823 gastric tumor-bearing mice	Gastric adenocarcinoma	Suppression tumor growth; Drug sensitivity; Inhibition of EMT	[97]

**Table 3:** Examples of NP-mediated toxicology by modulating EMT.

Entry	NP	Target Cell Line/Model	Tissue Type	Biological Effect	Refs
1	AgNPs	BEAS-2B	Bronchial	Induction in cell migration and invasion; Induction of mesenchymal phenotype; Collagene deposition; Alteration of transcriptomic profile; Induction of EMT; Inhibition of apopotisis	[209,210]
2	SiNPs + benzo[a]pyrene-7, 8-dihydrodiol-9, 10-epoxide (BPDE)	BEAS-2B THP-1	Bronchial; Blood	Activation of EMT; Inflammation	[211]
3	CeO_2_-NPs	Lung fibroblast Alveolar type II (ATII) cells	Lung	Activation of EMT; Cytotoxicity; Decreasing proliferation; Lung fibrosis	[212]
5	Carbon nanotubes	BEAS-2B Lung fibroblast Alveolar type II (ATII) cells MeT-5A, LP-9 A459	Bronchial; Lung; Pleural mesothelium	Induction in EMT Induction of Slug; Induction of TGFbeta/Smad; H-Ras/ERK; Akt/GSK-3β/SNAIL-1 Fibrosis; Oncogenic transformation; tumor progression; Metastasis; inflammation	[213,214,215,216,217]
6	TiO_2_-NPs	CD-1 (ICR) female mice	Kidney	Kidney injury, nephrotoxicity; Inflammation, fibrosis; Increasing in EMT and Wnt signaling	[218]
